# *Candida glabrata* Prostatitis: A Rare Case in a Patient With Type 2 Diabetes Mellitus on Dapagliflozin Treatment

**DOI:** 10.1155/crdi/7705938

**Published:** 2026-06-22

**Authors:** Elena Gloria Torres García, Cristina Marcelo Calvo, Paula Navarro-Carrera, Alejandro Díez-Vidal, Beatriz Díaz-Pollán

**Affiliations:** ^1^ Internal Medicine Department, La Paz University Hospital, Madrid, Spain, hulp.es; ^2^ Internal Medicine Department, Infectious Diseases Unit, IdiPAZ (La Paz Institute for Health Research), CIBERINFEC (Centro de Investigación Biomédica en Red de Enfermedades Infecciosas), Instituto de Salud Carlos III, La Paz University Hospital, Madrid, Spain, hulp.es; ^3^ Department of Clinical Microbiology and Parasitology, Hospital Universitario La Paz-H. Carlos III, Madrid, Spain

**Keywords:** *Candida glabrata*, candidemia, dapagliflozin, diabetes mellitus, fungal prostatitis, SGLT2 inhibitor, urinary tract infection

## Abstract

We present a case of acute *Candida glabrata* prostatitis complicated by secondary candidemia in a 72‐year‐old man with poorly controlled Type 2 diabetes mellitus and benign prostatic hyperplasia, without classical healthcare‐associated risk factors for invasive candidiasis. The patient presented with fever, urinary symptoms, and markedly elevated prostate‐specific antigen. Urine and blood cultures confirmed disseminated *C. glabrata* infection. Despite a comprehensive diagnostic workup, no alternative source of candidemia was identified, supporting primary prostatic infection with secondary hematogenous dissemination. Among the identified predisposing factors was ongoing treatment with dapagliflozin, a sodium–glucose cotransporter 2 inhibitor (SGLT2 inhibitor). While SGLT2 inhibitor use is established as a risk factor for superficial mycotic genitourinary infections, invasive fungal complications remain poorly characterized. This case suggests that the SGLT2 inhibitor–induced glucosuria, in combination with structural urinary obstruction and poorly controlled diabetes, may contribute to invasive fungal infections and highlights the need for clinical vigilance in patients with multiple converging risk factors.

## 1. Introduction

*Candida glabrata* prostatitis is an uncommon manifestation of invasive candidiasis, typically occurring in the context of significant immunosuppression or recent urological manipulation [1]. While invasive fungal infections are classically associated with profound immune dysfunction, severe genitourinary involvement may also occur in patients with metabolic comorbidities such as diabetes mellitus and structural urological abnormalities.

The widespread use of sodium–glucose cotransporter‐2 inhibitors (SGLT2 inhibitors) has introduced a new variable into this landscape. By inducing therapeutic glucosuria, these agents significantly alter the genitourinary microenvironment, increasing the risk of genitourinary infections [2]. However, the specific contribution of SGLT2 inhibitors to invasive fungal infection has not been well established [3].

This report presents a rare case of acute *C. glabrata* prostatitis and secondary candidemia in a patient receiving dapagliflozin. Given the increasing use of SGLT2 inhibitors, this case highlights the need to consider the potential for invasive fungal complications in this population, beyond the more frequently reported uncomplicated urinary tract infections.

## 2. Case Presentation

A 72‐year‐old man with poorly controlled Type 2 diabetes mellitus (T2DM), with an HbA1c of 8.4%, and a known history of benign prostatic hyperplasia (BPH) presented to the Emergency Department of a tertiary care hospital in Madrid, Spain, on November 2, 2024, with hypogastric pain, tenesmus, and dysuria. BPH had been documented approximately 5 years earlier based on mild lower urinary tract symptoms and ultrasound findings; however, the patient had not undergone regular urological follow‐up or specific treatment. His home medications included metformin, vildagliptin, and dapagliflozin (10 mg once daily, initiated two years prior).

Initial physical examination revealed no suprapubic fullness, and the patient was afebrile. He reported a 2‐week history of diarrhea without identifiable epidemiologic risk factors of infectious etiology. Urinalysis demonstrated numerous yeast forms, and he was discharged with oral cefuroxime and fluconazole.

Two days later, he returned with acute urinary retention, fever (39 °C), and persistent diarrhea although with decreased stool frequency. No fecal culture was performed. Laboratory analysis revealed leukocytosis with neutrophilia (Table 1), elevated C‐reactive protein (170 mg/L) and procalcitonin (1.1 ng/mL), prerenal acute kidney injury (serum creatinine 2.3 mg/dL), and an elevated prostate‐specific antigen (PSA) of 51 ng/mL. Urinalysis revealed abundant yeast forms and pyuria. Blood cultures were obtained, a bladder catheter was inserted, and empirical therapy with ceftriaxone was initiated. He was subsequently admitted to the Infectious Diseases ward.

**TABLE 1:** Evolution of key laboratory parameters from admission to discharge.

Parameter	At emergency department	At discharge	Reference range
Leukocytes (× 10^9^/L)	19.1	9.6	3.6–10.5
Neutrophils (× 10^9^/L)	10.2	5.2	1.5–7.7
Monocytes (× 10^9^/L)	1.7	1.3	0.1–0.9
C‐reactive protein (mg/L)	170	3.7	< 5
Procalcitonin (ng/mL)	1.1	0.1	< 0.5
Serum creatinine (mg/dL)	2.3	1.1	0.7–1.3
PSA (ng/mL)	51	14	< 4
Urinalysis	Yeast + pyuria	Cleared	Negative
Blood cultures	*Candida glabrata* (2/2)	Sterile after 48 h	Negative
Urine culture	*Candida glabrata*	—	Negative

Within 24 h, *C. glabrata* was isolated from both sets of peripheral blood culture (2/2) and the urine culture, confirming candidemia with a urinary source. No significant bacterial growth was detected in the urine culture. Antifungal therapy was escalated to liposomal amphotericin B (5 mg/kg/day).

An abdominopelvic CT scan revealed findings consistent with acute prostatitis (Figure 1), without evidence of other infectious foci. A subsequent urinary ultrasound confirmed grade IV BPH, which may have contributed by promoting urinary outflow obstruction and stasis.

**FIGURE 1:**
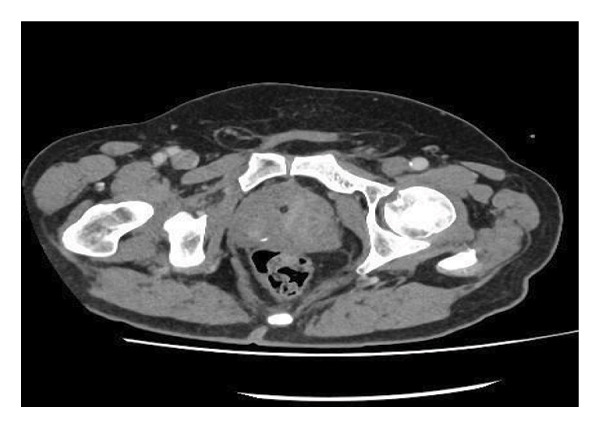
CT scan demonstrates marked prostatic enlargement with heterogeneous density and architectural distortion, consistent with acute prostatitis. Phlegmonous inflammatory changes are present, with no evidence of abscess formation.

A comprehensive diagnostic workup was performed to exclude systemic dissemination and identify potential sources or complications. Transthoracic echocardiography (TTE) and fundoscopic examination were normal, with no evidence of endocarditis or endophthalmitis.

Given persistent chronic monocytosis without an identifiable reactive cause, a bone marrow biopsy was performed to exclude chronic myelomonocytic leukemia (CMML) as a potential underlying immunosuppressive condition. Additionally, a colonoscopy was conducted to exclude a gastrointestinal portal of entry, and a PET‐CT scan showed no further metastatic or occult infectious foci.

While renal function initially improved with fluid resuscitation, it subsequently deteriorated during amphotericin B therapy. Treatment was switched to a combination of micafungin (100 mg/day) and fluconazole (400 mg/day), after which renal function recovered. Antifungal susceptibility testing confirmed the *C. glabrata* isolate was susceptible to fluconazole.

The patient remained afebrile, blood cultures cleared after 48 h of treatment, and inflammatory markers progressively improved (Table 1). Diarrhea resolved within the first 24 h of admission. PSA levels decreased to 14 ng/mL. After 10 days of intravenous treatment, he was discharged with oral fluconazole for a 4‐week course. Dapagliflozin was discontinued, and insulin therapy was initiated for glycemic control. A chronological summary of the patient’s clinical course is shown in Table 2.

**TABLE 2:** Clinical timeline, microbiological findings, imaging results, and treatment.

Time point	Clinical findings	Microbiology	Imaging	Management
Initial Emergency Department visit	Hypogastric pain, tenesmus, dysuria. Afebrile. Two‐week history of diarrhea. Known T2DM (HbA1c 8.4%) and untreated BPH.	Urinalysis: abundant yeast forms.	—	Discharged with oral cefuroxime and fluconazole. Home medications included metformin, vildagliptin, and dapagliflozin.
Day 2: readmission	Acute urinary retention and fever (39°C). Persistent diarrhea. Leukocytosis with neutrophilia, CRP 170 mg/L, procalcitonin 1.1 ng/mL. Prerenal AKI (creatinine 2.3 mg/dL).	Urinalysis: yeast forms and pyuria. Blood cultures obtained.	—	Bladder catheter inserted. Empirical ceftriaxone started. Hospital admission.
Within 24 h	Clinical suspicion of invasive fungal infection.	*Candida glabrata* isolated from urine culture and from 2/2 peripheral blood cultures.	—	Liposomal amphotericin B initiated (5 mg/kg/day).
Diagnostic evaluation	Investigation of primary vs. secondary focus of infection.	—	CT scan: findings consistent with acute prostatitis without abscess. Ultrasound: grade IV BPH. TTE and fundoscopy: normal. PET‐CT and colonoscopy: no alternative focus.	Continuation of antifungal therapy.
Hospital course	Initial renal improvement followed by amphotericin‐associated renal deterioration.	Follow‐up blood cultures sterile after 48 h.	—	Antifungal therapy switched to micafungin plus fluconazole.
Outcome and discharge	Afebrile. Inflammatory markers improved. Diarrhea resolved. Recurrent urinary retention requiring catheter.	Blood cultures negative.	—	Discharged after 10 days of IV therapy with oral fluconazole for 4 weeks. Insulin initiated and dapagliflozin discontinued.

## 3. Discussion

*Candida spp*. are commensal fungi belonging to the phylum Ascomycota that commonly colonize the skin, oral cavity, and the gastrointestinal and genitourinary tracts. Under specific conditions, they can act as opportunistic pathogens, causing infection through direct invasion or hematogenous dissemination, particularly in hospitalized or immunocompromised populations [4].

Invasive candidemia is typically associated with well‐recognized risk factors such as immunosuppression, parenteral nutrition, broad‐spectrum antibiotics, central venous catheterization, and underlying comorbidities such as diabetes mellitus [1, 4]. In this case, poorly controlled T2DM was the primary predisposing factor. Candidemia may represent either a primary infection or secondary dissemination. While bacterial prostatitis usually results from an ascending urinary tract infection, fungal prostatitis is more frequently associated with hematogenous seeding [5]. However, in our patient, no alternative source of candidemia was identified despite an extensive diagnostic workup, making primary prostatitis with secondary hematogenous dissemination the most likely scenario. Although the recent history of diarrhea raises the possibility of gastrointestinal translocation of *C. glabrata*, this hypothesis could not be confirmed in the absence of intestinal lesions or overt immunosuppression, as evidenced by negative colonoscopy and bone marrow biopsy.

Acute primary prostatitis is predominantly bacterial, typically caused by Enterobacteriaceae [6]. Fungal prostatitis is rare and usually occurs in patients with the same risk factors for candidemia. Among Candida species, *C. albicans* remains the most frequently isolate, followed by *C. glabrata* and *Pichia kudriavzevii* (*C. krusei*) although other fungal pathogens such as *Aspergillus fumigatus*, *Cryptococcus neoformans*, *Coccidioides immitis*, and *Histoplasma capsulatum* have also been reported [7].

SGLT2 inhibitors, such as dapagliflozin, provide significant cardiovascular and renal benefits but induce therapeutic glucosuria, which may facilitate microbial growth in the urinary tract [8]. Evidence regarding their association with urinary tract infections remains inconsistent, with previous meta‐analyses reporting heterogeneous results [2, 3]. A Spanish pharmacovigilance report (FEDRA, 2013–2017) documented 159 genitourinary infections associated with SGLT2 inhibitor therapy, including 11 hospitalizations and 1 fatal outcome [9]. Nevertheless, most infections related to SGLT2 inhibitors are superficial genital mycoses rather than invasive diseases [9, 10].

Several cases of *C. glabrata* urinary tract infections have been reported in patients receiving SGLT2 inhibitor therapy [11–15]. As summarized in Table 3, most occurred in individuals with significant predisposing factors such as structural urinary tract abnormalities, prior urological surgery, or immunosuppression. For instance, a recent case series of oncological patients described a severe fungal urinary tract infection caused by *C. glabrata* in a patient with urothelial carcinoma who had undergone cystoprostatectomy and pelvic lymphadenectomy and was receiving chemotherapy with carboplatin and gemcitabine, highlighting the role of profound immunosuppression and previous urological surgery as contributing factors [16]. In contrast, our patient had no history of recent urological manipulation or chemotherapy‐related immunosuppression. Notably, none of the previously reported cases involved acute prostatitis complicated by candidemia.

**TABLE 3:** Comparative characteristics of reported cases of *Candida glabrata* urinary tract infection in patients receiving SGLT2 inhibitor.

Variable	Cases‐corona, 2020	Woloshuk, 2021	Sahara, 2025	Zeelenberg, 2023	Posado‐domínguez, 2023	Present case
Age/Sex	66 F	67 F	69 F	72 M	74 M	72 M
SGLT2 inhibitor	Empagliflozin	Empagliflozin 25 mg	SGLT2 inhibitor	Dapagliflozin	Canagliflozin	Dapagliflozin 10 mg
Duration of therapy	Not specified	11 months	Not specified	Not specified	Not specified	2 years
Diabetes control	Not specified	HbA1c 7.9%	Not specified	HbA1c 10.8%	Not specified	HbA1c 8.4%
Structural abnormality	Kidney transplant	Ureteral stone + hydronephrosis	Not specified	None	Prior cystoprostatectomy (urothelial carcinoma)	Grade IV BPH
Immunosuppression	Renal transplant	No	Chemotherapy (breast cancer)	No	Chemotherapy (carboplatin + gemcitabine)	No (CMML excluded)
Clinical presentation	Emphysematous pyelonephritis	Fungal bezoar + pyelitis	Pyelonephritis + bacteremia	Chronic cystitis	Severe fungal UTI	Acute prostatitis
Blood cultures	*Candida glabrata*	*Candida glabrata*	*Candida glabrata*	No candidemia	Not reported	*Candida glabrata* (2/2)
Urine culture	*Candida glabrata*	*Candida glabrata*	*Candida glabrata*	*Candida glabrata*	*Candida glabrata*	*Candida glabrata*
Antifungal therapy	Micafungin	Amphotericin B ⟶ micafungin	Micafungin ± fluconazole	None (drug withdrawal only)	Anidulafungin	Amphotericin B ⟶ micafungin + fluconazole
Surgical intervention	No	Ureteroscopic removal	No	Transurethral resection of the bladder (biopsy)	No	No
Outcome	Recovery	Recovery	Recovery	Resolution after dapagliflozin withdrawal	Recovery	Recovery
Prostatitis	No	No	No	No	No	Yes
Classical risk factors for candidemia present	Yes (renal transplant)	Yes (urinary obstruction + instrumentation)	Yes (chemotherapy)	No	Yes (chemotherapy + urologic surgery)	No

Beyond urinary tract infections, *C. glabrata* has also been implicated in a case of Fournier’s gangrene in a patient taking SGLT2 inhibitor [16]. The emergence of *C. glabrata* as a relevant pathogen is particularly concerning due to its increasing rates of reduced fluconazole susceptibility [17].

In this patient, poorly controlled T2DM, SGLT2 inhibitor therapy, and urinary outflow obstruction with associated stasis—likely related to underlying BPH—may have acted synergistically to predispose him to invasive disease.

Regarding management, empirical therapy for *C. glabrata* candidemia typically requires an echinocandin or amphotericin B, until susceptibility data are available [18]. For urinary tract infections caused by *C. glabrata*, fluconazole or amphotericin B is commonly recommended.

In our case, liposomal amphotericin B was initially used but was discontinued due to nephrotoxicity. Therapy was then switched to a combination of micafungin and fluconazole, as described in other reported cases [11]. This approach was chosen because micafungin has limited prostatic penetration, while the isolate’s azole susceptibility was initially unknown. Once fluconazole susceptibility was confirmed, treatment was de‐escalated to fluconazole monotherapy, which effectively targeted both the bloodstream and the prostatic focus.

The recommended duration of therapy for candidemia is at least 2 weeks after blood culture clearance although longer treatment is advised when deep organ involvement is present [19]. In bacterial prostatitis, treatment typically ranges from two to four weeks to prevent chronicity [20]. Given the rarity of Candida prostatitis, the duration of antifungal therapy was extrapolated from treatment recommendations for bacterial prostatitis.

## 4. Conclusion

Although Candida urinary tract infections have been described in patients receiving SGLT2 inhibitors, to our knowledge, this is the first reported case of acute *C. glabrata* prostatitis complicated by secondary candidemia in a patient without classical healthcare‐associated risk factors or prior urological manipulation.

This case highlights the potential role of SGLT2 inhibitor–induced glucosuria as a contributing factor in complicated fungal infections, particularly when combined with conditions promoting urinary stasis, such as urinary outflow obstruction related to BPH. It also underscores the increasing clinical relevance of *C. glabrata* and other nonalbicans Candida species in invasive genitourinary infections, as well as the diagnostic and therapeutic challenges associated with these pathogens.

Further studies are required to clarify the relationship between SGLT2 inhibitors and invasive fungal genitourinary infections.

**Nomenclature**
SGLT2 inhibitor: Sodium–glucose cotransporter 2 inhibitor
T2DM: Type 2 diabetes mellitus
BPH: Benign prostatic hyperplasia
TTE: Transthoracic echocardiography
CMML: Chronic myelomonocytic leukemia
FEDRA: Spanish Pharmacovigilance Adverse Reaction Data

## Funding

This research received no external funding.

## Disclosure

Preprint statement: This manuscript has not been previously published and has not been posted as a preprint.

Conference presentation: This work was partially presented as a poster at the SEMI (Spanish Society of Internal Medicine) Congress 2025, from 26 to 28 November 2025. The present manuscript includes additional data, a literature review (Table 3), and an expanded discussion not included in the original abstract.

## Ethics Statement

Ethical approval was not required for this case report according to institutional policies.

Reporting guidelines: This case report was prepared in accordance with the CARE (Case Report) reporting guidelines.

## Consent

The patient provided oral consent for the publication of clinical details and imaging data.

## Conflicts of Interest

The authors declare no conflicts of interest.

## Data Availability

All data relevant to this case report are included within the article.
